# Novel Application of an Ultrasonic Bone Aspirator for Endoscopic Modified Medial Maxillectomy

**DOI:** 10.3389/fsurg.2022.870380

**Published:** 2022-06-01

**Authors:** Hiromasa Takakura, Hirohiko Tachino, Yutaro Oi, Tram Anh Do, Hideo Shojaku

**Affiliations:** Department of Otorhinolaryngology, Head and Neck Surgery, Faculty of Medicine, Academic Assembly, University of Toyama, Toyama, Japan

**Keywords:** endoscopic modified medial maxillectomy, intraoperative complication, maxillary sinus, nasolacrimal duct, ultrasonic bone aspirator

## Abstract

**Background:**

Endoscopic modified medial maxillectomy (EMMM) is a surgical technique developed to approach maxillary sinus lesions, such as papilloma and postoperative maxillary cyst, while preserving the postoperative nasal morphology and nasal function. In this technique, a diamond burr is used to remove the bone, which may damage adjacent soft tissue. We developed EMMM using an ultrasonic bone aspirator (UBA) instead of a conventional diamond burr. The purpose of this study was to clarify the effectiveness of the UBA in EMMM in comparison to the conventional diamond burr technique in terms of operative time, intraoperative complications, postoperative symptoms, and recurrence.

**Methods:**

The medical records of all patients who underwent EMMM at Toyama University Hospital between June 2014 and December 2021 were reviewed. Patients who met the inclusion criteria were separated into Group 1, in which the UBA was used for EMMM, and Group 2, in which a drill with a diamond burr was used. Data on patient demographics, operation time, frequency of intraoperative complications and postoperative symptoms, and recurrence were statistically compared between the two groups.

**Results:**

There were no significant differences between the two groups in the patient demographic data, operative time, postoperative symptoms, or frequency of recurrence. The frequency of intraoperative damage to adjacent soft tissues was significantly lower in patients who underwent EMMM with the UBA in comparison to those who underwent EMMM with a conventional diamond burr.

**Conclusion:**

The application of the UBA to EMMM can improve surgical safety and facilitate surgical procedures.

## Introduction

Endoscopic medial maxillectomy (EMM), first reported by Kamel in 1995 (1), is one of the surgical techniques used to address poor access to the maxillary sinus (MS) and it currently remains a relevant technique in the treatment of various MS diseases where standard medial meatus antrostomy or other techniques are deemed inadequate (2), especially inverted papilloma (3, 4). EMM involves resection of the inferior turbinate (IT), lateral wall of the nasal cavity, and/or the nasolacrimal duct (NLD), resulting in many disadvantages, including persistent crusting, warming or humidifying dysfunction of inspired air, epiphora, and/or dacryocystitis (5). To improve these disadvantages, EMM with preservation of the IT and NLD, named endoscopic modified medial maxillectomy (EMMM) (6, 7) or modified transnasal endoscopic medial maxillectomy (5, 8), has recently been developed and is reported to be useful not only for papilloma in the MS (6, 9) but also for the treatment of postoperative maxillary cyst (PMC; defined as mucocele of the MS after a Caldwell–Luc operation (6) or odontogenic cyst or tumor (7)). In EMMM, osteotomy of the IT, NLD, and medial wall of the MS is performed with a chisel or diamond burr (6), and the surgeon must have a solid skill level to use these instruments because they have the potential to damage soft tissue around the bone, such as the nasal mucosa, NLD, or maxillary lesions.

An ultrasonic bone aspirator (UBA) (Sonopet UST2001, Stryker, Kalamazoo, MI, USA) is a device with an oscillating metal tip that can perform osteotomy using vibration with both longitudinal and reciprocating (sinusoidal) torsional movements at an ultrasonic frequency of 25 kHz (10). The mechanism of the UBA is based on piezoelectric technology (11). Neither the tip nor the shaft rotates 360° like a typical high-speed drill, making it less likely to entrap soft tissues around the bone (10). We have developed the EMMM technique utilizing the UBA, which reduces intraoperative soft tissue damage and makes the operation safer and easier. In this study, we attempted to clarify the usefulness of the UBA by retrospectively comparing data from patients who underwent EMMM with the UBA and those who underwent EMMM with a conventional drill.

## Surgical Technique

**Figure 1:**
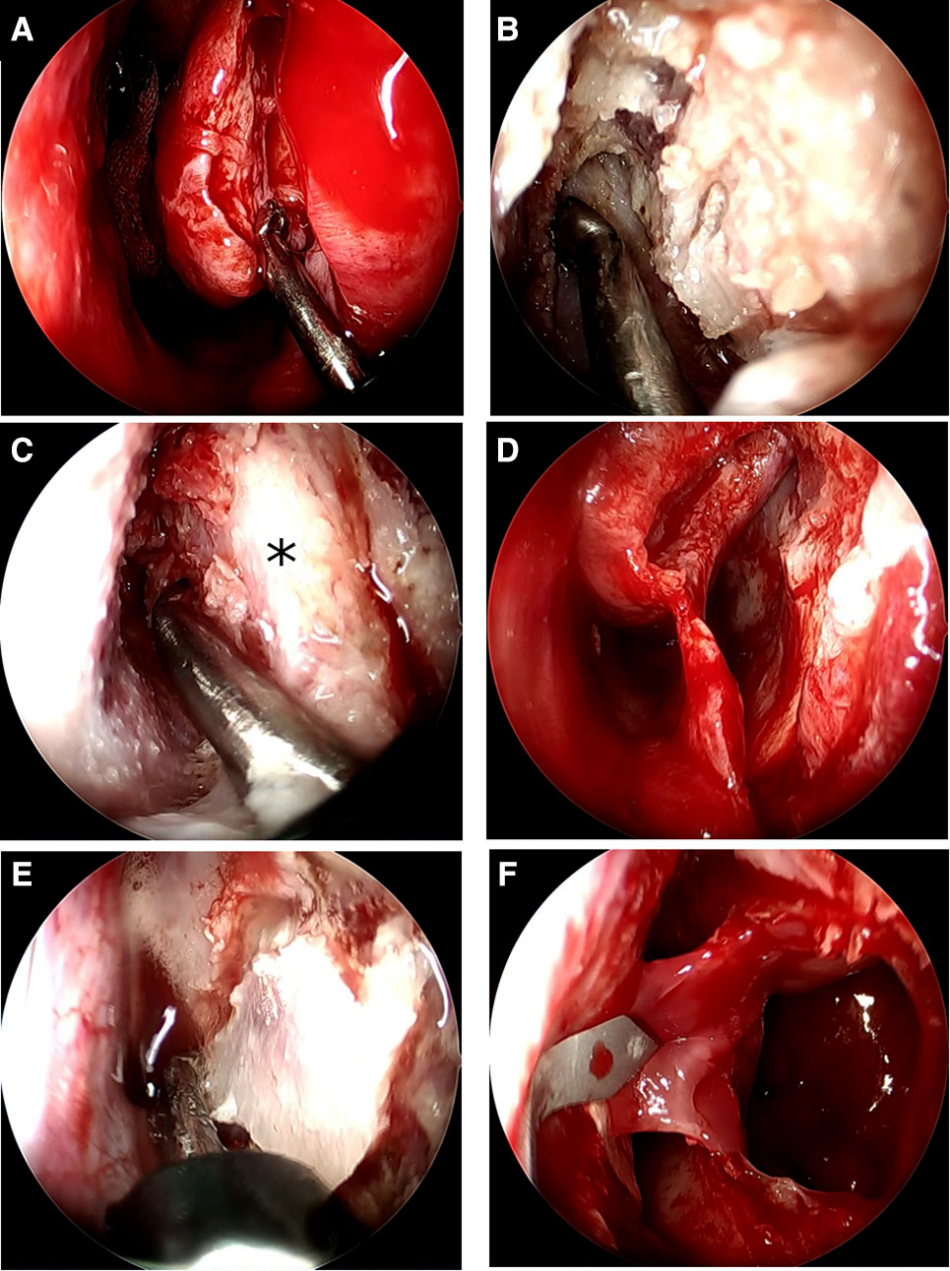
The endoscopic intraoperative findings of the left nasal cavity. (**A**) A mucosal incision was made from the anterior part of the lateral nasal wall to the bottom of the nasal cavity parallel to the mucocutaneous junction, behind the pyriform aperture, and the metal tip of the UBA was set on the attached portion of the IT bone. (**B**) Cutting of the IT bone attached to the lateral nasal wall proceeds upward to identify the NLD. (**C**) The UBA can crush the bone around the nasolacrimal duct without damaging the surrounding soft tissue. *=NLD. (**D**) The mucosa of the IT and the NLD was elevated and displaced medially from the lateral wall of the nasal cavity. (**E**) The bone on the outer wall of the nasal cavity is cut as needed with the UBA. Using brush-like strokes on the area to be cut without pressing hard, the UBA can cut without damaging the soft tissues at the back. (**F**) The MS mucosa can then be incised with a mucosal scalpel to gain access to the lesion within the MS.

EMMM was performed under general anesthesia in all patients. A mucosal incision was made from the anterior part of the lateral nasal wall to the bottom of the nasal cavity parallel to the mucocutaneous junction behind the pyriform aperture (Figure 1A). The nasal mucosa was elevated medially from the lateral wall of the nasal cavity, including the mucosa of the IT, and then, the IT bone was visualized. We used the UBA to cut the conchal crest instead of a chisel or diamond bur in conventional EMMM. The tip of the UBA was set under the attached portion of the IT bone and we cut upward to identify the NLD (Figure 1B). The inferior half of the IT was preserved. Due to the characteristics of the UBA, there is little chance of involving the surrounding mucosa or damaging the soft tissue behind the bone (Figure 1C), so the NLD could be easily exposed by lightly pressing it against the bone. Then, the nasal mucosa and NLD were moved medially (Figure 1D). In the conventional EMMM procedure, the inner wall of the MS is shaved with a diamond burr for entry into the MS and approach to the maxillary lesion. We used the UBA for the operation instead of a diamond burr. When the UBA was pressed lightly against the inner bony wall of the MS or lesion, it was possible to remove the bone only in that area (Figure 1E). If we did not press it too hard, the mucosa or soft tissue lesions behind the bony wall were unlikely to be damaged. After cutting the bone around the area to be opened, the bone fragments could be carefully detached to expose the medial mucosa of the MS without causing significant damage. The MS mucosa can then be incised with a mucosal scalpel to gain access to the lesion within the MS (Figure 1F). If the opening was to be enlarged, additional bone from the medial wall was resected with the UBA. When we had to drill the posterior part of the pyriform aperture away to visualize and manipulate the anterior wall of the MS from which the papilloma originated, we used a 70° diamond burr, as only straight-type UBAs are available. After completion of the manipulation of the MS, the IT and NLD were laterally replaced, and the incised mucosa of the IT was sewn to the lateral nasal wall.

## Methods

The medical records of all patients who underwent EMMM at Toyama University Hospital between June 2014 and December 2021 were reviewed. Inclusion criteria consisted of any patient who received EMMM for any lesion of the MS or maxilla. In this study, we determined that a follow-up period of at least 3 months (12 weeks) for postoperative symptoms was necessary. Therefore, patients who could not be followed up for more than 3 months were excluded from this study. Patients who met the criteria were separated into Group 1, in which the UBA was used for EMMM, and Group 2, in which a drill with a diamond burr was used.

All surgical procedures were performed by one senior surgeon (H. Tak.) and all patients were followed up at the outpatient clinic of the Department of Otorhinolaryngology, Toyama University Hospital by two doctors (H. Tak. and H. Tac.). Since the UBA instrument is shared by several departments in our hospital, we used the UBA when it was available for EMMM and the diamond burr when other departments were using the UBA. We did not intentionally use the UBA for specific diseases. However, for PMCs located on the outer side of the maxilla, where the tip of the UBA could not physically reach, a curved diamond burr was selected from the start of the procedure instead of the UBA. Data on patient demographics and outcome measures including operation time from the mucosal incision of the IT to completion of exposure of the MS mucosa or lesion, intraoperative complications, postoperative symptoms, and presence of recurrence were collected retrospectively. Intraoperative complications consisted of bleeding from osteotomy or soft tissue injury, MS mucosal (or cyst wall) injury, NLD injury, and nasal mucosal injury. Intraoperative bleeding was defined as bleeding from the cutting surface of the bone or surrounding soft tissues during bone removal by each device, requiring some form of hemostatic manipulation (i.e., hemostasis by electrocoagulation or compression with gauze soaked in epinephrine). The intraoperative MS mucosal (or lesion) injury was defined as direct damage, penetration, or bleeding of the soft tissues by the device during bone removal. Intraoperative NLD injury was defined not only as penetration of the soft NLD during osteotomy and leakage of tear fluid but also included bleeding from the NLD due to contact with each device. The nasal mucosal injury was defined as damage, bleeding, or penetration of nasal mucosa due to direct contact or entrapment of each device. Surgical time and intraoperative complications were judged by two otolaryngologists different from the surgeon (H. Tak.), who watched videos of the operation independently. In case of disagreement, these two otolaryngologists consulted each other, and the final decision was made by consensus. Postoperative symptoms consisted of epiphora, bleeding, pain, facial swelling, and numbness. Postoperative bleeding was defined as bleeding from a surgical wound that required hemostatic repacking or hemostatic surgery 24 h to 10 days after surgery. Patients who experienced postoperative pain and numbness that lasted for more than 3 months were considered to be positive for postoperative pain and numbness, respectively. The presence of facial swelling was determined by the subjective judgment of the surgeon in addition to the patient’s awareness of the symptom.

This study was approved by the Ethics Committee of the Toyama University Hospital, Toyama, Japan (approval number: R2020166).

## Statistical Analyses

Among the patient demographics and outcome measures, quantitative variables are presented as the mean ± standard deviation, while categorical variables are presented as the number and percentage. Binary outcomes were compared using the χ^2^ test or Fisher’s exact tests. Comparisons between the two groups were performed using the Student’s *t*-test or the Mann–Whitney test depending on the distribution of variables according to the Shapiro–Wilk test and the homogeneity of variance according to the Levene test. *P*-values of <0.05 were considered to indicate statistical significance. Statistical analyses were performed using the SPSS Statistics software program (version 26.0; IBM Corporation, Chicago, IL).

## Results

Thirty-six patients who underwent EMMM in Toyama University Hospital between June 2014 and December 2021 were included. All of these patients could be followed up for more than 3 months postoperatively; thus, 36 patients were enrolled in this study. All of these had their medical records and surgical videos available for review. Twenty-four patients received EMMM with the UBA (Group 1), while 12 patients received EMMM with a conventional drill (Group 2). The demographic data of these two groups are shown in Table 1. Group 1 included 17 (70.8%) male patients and 7 (29.2%) female patients, while Group 2 included 8 (66.7%) male patients and 4 (33.3%) female patients. The mean age of the patients was 61.1 ± 15.6 years in Group 1 and 61.3 ± 15.4 years in Group 2. Regarding the laterality of the operation, 12 patients (50.0%) had a right-sided operation and 12 patients had a left-sided operation in Group 1. In Group 2, 6 patients (50.0%) had a right-sided operation and 6 had a left-sided operation. Regarding the primary disease, in Group 1, 11 patients (45.8%) had PMC, 7 (29.2%) had papilloma originating from the MS, 3 (12.5%) had maxillary sinusitis, 2 (8.3%) had a foreign body (spicule, *n* = 1; ectopic tooth, *n* = 1), and 1 (4.2%) had maxillary carcinoma. In Group 2, 6 patients (50.0%) had PMC, 3 (25.0%) had papilloma originating from the MS, 1 (8.3%) had maxillary sinusitis, and 2 (16.7%) had a hematoma in the MS. The follow-up period was 13.8 ± 12.9 months in Group 1 and 8.5 ± 6.2 months in Group 2. There were no significant differences between the two groups with regard to sex (*P* = 0.544, Fisher’s exact test), age (*P* = 0.964, Student’s *t*-test), laterality (*P* = 0.141, Fisher’s exact test), primary disease (*P* = 0.635, χ^2^ test), or follow-up period (*P* = 0.585, Mann–Whitney *U* test).

**Table 1:** Demographic data of Groups 1 and 2.

	Group 1	Group 2	*P*-value	Statistics
	(*n* = 24)	(*n* = 12)		
Sex (M/F)	17 (70.8)/7	8 (66.7)/4	0.544	Fisher
Age (year)	61.1 ± 15.6	61.3 ± 15.4	0.964	*t*-test
Side (Right/Left)	12 (50.0)/12	9 (75.0)/3	0.141	Fisher
Primary disease				
PMC	11 (45.8)	6 (50.0)	0.635	*χ* ^2^
Others	13 (54.2)	6 (50.0)		
Papilloma	7	3		
Sinusitis	3	1		
Foreign body	2			
Other tumors	1	2		
	Carcinoma, Biopsy	Hematoma		
Follow-up (months)	13.8 ± 12.9	8.5 ± 6.2	0.585	Mann–Whitney *U*

*Data are presented as the mean ± standard deviation, (percentage) or number*.

*PMC, postoperative maxillary cyst; Fisher, Fisher’s exact test; t-test, Student’s t-test; χ^2^, χ^2^ test; Mann–Whitney U, Mann–Whitney U test*.

Table 2 compares the operation time and intraoperative complications between the two groups. The operation time from the onset to the completion of exposure of the maxillary mucosa or lesion was 31.4 ± 18.0 min in Group 1 and 33.3 ± 16.6 min in Group 2. Intraoperative bleeding was found in 4 of 24 patients (16.7%) in Group 1 and 6 of 12 patients (50.0%) in Group 2. Injury of the maxillary mucosa was found in 2 of 24 patients (8.3%) in Group 1 and 9 of 12 patients (75.0%) in Group 2. Injury of the NLD was found in 0 of 24 patients (0.0%) in Group 1 and 5 of 12 patients (41.7%) in Group 2. Injury of the nasal mucosa was found in 0 of 24 patients (0.0%) in Group 1 and 5 of 12 patients (41.7%) in Group 2. There was no significant difference between the two groups in operative time (P = 0.768, Student’s *t*-test); however, the frequency of intraoperative bleeding (P = 0.045, Fisher’s exact test), MS mucosal injury (*P = 0.0001*, Fisher’s exact test), NLD injury (P = 0.002, Fisher’s exact test), and nasal cavity mucosal injury (P = 0.002, Fisher’s exact test) was significantly lower in Group 1 than in Group 2.

**Table 2:** Operation time and intraoperative complications.

	Group 1	Group 2	*P*-value	Statistics
	(*n* = 24)	(*n* = 12)		
Operation time (minutes)	31.4 ± 18.0	33.3 ± 16.6	0.768	*t*-test
Intraoperative complications (positive/negative)				
Bleeding	4 (16.7)/20	6 (50.0)/6	0.045*	Fisher
Injury of maxillary mucosa (or lesion)	2 (8.3)/22	9 (75.0)/3	0.0001*	Fisher
Injury of nasolacrimal duct	0 (0.0)/24	5 (41.7)/7	0.002*	Fisher
Injury of nasal mucosa	0 (0.0)/24	5 (41.7)/7	0.002*	Fisher

*Data are presented as the mean ± standard deviation, (percentage) or number*.

*Fisher, Fisher’s exact test; t-test, Student’s t-test.*

Table 3 shows the frequency of postoperative symptoms in two groups. Epiphora was found in 5 of 24 patients (20.8%) in Group 1 and 1 of 12 patients (8.3%) in Group 2. In all cases, epiphora improved after the removal of nasal packing. Postoperative bleeding was found in 1 of 24 patients (4.2%) in Group 1 and 1 of 12 patients (8.3%) in Group 2. Facial swelling was found in 6 of 24 patients (25.0%) in Group 1 and 1 of 12 patients (8.3%) in Group 2, and it improved and disappeared within one month after surgery. Facial numbness lasting more than 3 months after surgery was found in 3 of 24 patients (12.5%) in Group 1 and 1 of 12 patients (8.3%) in Group 2. Two of the three patients in Group 1 were left with permanent facial numbness. The remaining patient in Group 1 and one patient in Group 2 showed spontaneous resolution of numbness 4 months after surgery. Postoperative pain lasting more than 3 months after surgery was found in 1 of 24 patients (4.2%) in Group 1 and 1 of 12 patients (8.3%) in Group 2. These two patients showed resolution of their postoperative pain 4 months after surgery. Recurrent disease was found in 1 papilloma patient among the 24 patients (4.2%) in Group 1 and 1 PMC patient among the 12 patients (8.3%) in Group 2. There were no statistically significant differences in the postoperative symptoms of Groups 1 and 2.

**Table 3:** Postoperative symptoms and recurrence.

	Group 1	Group 2	*P*-value	Statistics
	(*n* = 24)	(*n* = 12)		
Postoperative symptoms (positive/negative)				
Epiphora	5 (20.8)/19	1 (8.3)/11	0.331	Fisher
Bleeding	1 (4.2)/23	1 (8.3)/11	0.562	Fisher
Facial swelling	6 (25.0)/18	1 (8.3)/11	0.235	Fisher
Facial numbness	3 (12.5)/21	1 (8.3)/11	0.593	Fisher
Pain	1 (4.2)/23	1 (8.3)/11	0.562	Fisher
Recurrence	1 (4.2)/23	1 (8.3)/11	0.562	Fisher
	(papilloma)	(PMC)		

*Data are presented as (percentage) or number.*

*PMC, postoperative maxillary cyst; Fisher, Fisher’s exact test.*

## Discussion

The UBA device transforms electrical energy into mechanical energy, causing the various tips to vibrate in the ultrasonic band of approximately 25–30 kHz (11). At this frequency of vibration, the UBA can cut bone but causes little damage to soft tissue (11). The UBA’s tissue removal mechanism uses high-frequency vibration to break hydrogen bonds and denature proteins in the tissue. The tissue is then emulsified by the formation of vapor bubbles and quickly washed away by the attached irrigation system (11). In addition to its soft tissue sparing properties, in comparison to surgery with conventional drills, UBA piezosurgery has also been proven to result in better bone healing after surgery and a decreased level of inflammatory cells at the operative site (11).

The UBA has been used in a wide range of fields, including skull base surgery (12–14), orbital and lacrimal sac surgery (15–17), and spine surgery (18–21). In the field of otorhinolaryngology, the UBA has been used for facial nerve decompression surgery (10), frontal sinus osteoma removal (22–24), transcanal endoscopic ear surgery (25), extended frontal sinusotomy (26), septoplasty and inferior turbinectomy (27, 28), endoscopic posterior split and cartilage graft laryngoplasty (29), retrosigmoid craniotomy (30), and septorhinoplasty (31–33). However, no previous studies have described the application of the UBA in EMMM.

Some studies have reported the comparison of safety and efficacy between the UBA and a conventional drill. Cho et al. conducted a retrospective study to compare the efficacy and safety of the UBA for lateral orbital decompression in thyroid eye disease between a group (18 patients) treated using a UBA and a group (*n* = 18) treated using a high-speed drill with a cutting burr (34). They concluded that the UBA was superior to conventional instruments in terms of its ease of use, reduced need for removal of the lateral orbital rim, slightly shorter operative time, and reduced risk of dural violation causing spinal fluid leakage. Massey et al. retrospectively compared nine cases of extended endoscopic frontal sinusotomy performed using the UBA with nine cases performed using a conventional high-speed drill (26). They noted that the UBA group had more malignant neoplasms, papillomas, and Draf III procedures in comparison to the conventional drill group and that there were no significant differences in postoperative symptoms or outcomes between the two groups. They also noted that less damage to surrounding tissues and effective clearance of blood and debris from the surgical field are advantages of the UBA in extended frontal sinusotomy. Baddour et al. conducted a prospective, randomized controlled trial comparing the use of the UBA and conventional instruments in endoscopic transsphenoidal approaches in terms of operative time and blood loss and found that the operative time was significantly shorter and blood loss was significantly lower in the UBA group (35).

The results of our study showed that in EMMM, the UBA did not shorten the operative time; however, its use was associated with significantly less intraoperative bleeding or less intraoperative damage to the MS mucosa (lesion), the NLD, and the nasal cavity mucosa. In addition, we found that postoperative symptoms and treatment outcomes (i.e., recurrence) were comparable to those in patients treated using conventional devices. These results were generally consistent with those of previous comparative studies, and it was thought that the lack of rotational moment and the characteristic of not involving the surrounding soft tissue of the UBA were responsible for the characteristic of not causing damage to the surrounding soft tissue during bone drilling. Treatment with the UBA is associated with less intraoperative damage to the soft tissue, which we believe is beneficial to all surgeons but especially to beginners or residents with little surgical experience in the performance of EMMM.

Several previous studies have reported the outcomes and complications of conventional EMMM or similar surgical procedures. Nakayama et al. (6) reported one case of epiphora, one case of buccal swelling, and one case of postoperative bleeding among 15 patients. Wang et al. (36) reported no postoperative complications in seven cases. Suzuki et al. (8) reported that among 51 patients, 7 patients (13.7%) experienced numbness around the upper lip, 1 patient (2.0%) experienced numbness of the cheek, and 1 patient (2.0%) experienced cheek swelling. Nakayama et al. (37) reported numbness of the face and teeth in 1 of 27 patients. Only two studies mentioned intraoperative complications, both reported that no intraoperative complications (e.g., NLD injury) occurred (7, 37). In our study population, the frequency of intraoperative complications and postoperative symptoms tended to be higher in comparison to these reports. We believe that the higher frequency of complications in our study may be due to the more rigorous evaluation of intraoperative complications and postoperative symptoms, which were clearly defined.

There are several points to be noted when performing EMMM using the UBA. First, the field of view of the endoscope is impaired due to the mist emitted when using the UBA. It is necessary to use an irrigation system to ensure a clear endoscopic view at all times. Second, when using the UBA in a narrow surgical field, interference between the metal tip of the UBA and the endoscope may damage the outer cylinder of the endoscope and generate metal powder. By ensuring that the perfusion sheath of the UBA is always visible in the endoscopic field of view, contact between the endoscope and the metal tip can be prevented. Third, there is a hole for water suction at the back of the metal tip of the UBA, which may aspirate the detached mucosa and other soft tissues. It is advisable to keep a sufficient distance between the soft tissue and the suction hole or to place a small piece of paper on the mucosa to prevent damage caused by suction. Fourth, the UBA handpiece is only available in a straight type and not in a curved type, which may make it difficult to approach lesions in the outermost part of the maxilla. In such cases, we have to use a curved diamond burr.

We consider the following situations to be indications for UBA rather than a diamond burr in EMMM: (1) procedures performed by inexperienced surgeons; (2) cases involving malignant tumor or papilloma in the MS where the dissemination of tumor cells should be prevented; and (3) cases in which the working space between the lesion and the surrounding structures is narrow. On the other hand, we also believe that a diamond burr should be used for deep or lateral lesions of the maxilla that the UBA cannot reach.

The present study is associated with some limitations, including its retrospective design, and the relatively small number of cases that received EMMM using a conventional drill. It is possible that the UBA was preferred for more difficult cases because it is easier and performs more secure surgery, which may have led to bias in case selection. In our study, there was no significant difference in the operative time; however, we cannot deny the possibility that this bias may have affected the results. To evaluate the efficacy of the UBA in EMMM more accurately, a prospective study should be undertaken to compare EMMM with a UBA and EMMM with a conventional drill.

## Conclusion

We developed a new technique for the treatment of MS lesions in which a UBA was applied in EMMM, which preserved the IT and NLD. Our retrospective study revealed that the application of the UBA in EMMM significantly reduced the frequency of intraoperative bleeding and soft tissue injury in comparison to the conventional high-speed drill, and the postoperative symptoms and outcomes were comparable to those of the conventional technique. The characteristics of the UBA, including the absence of rotational moment and soft tissue entrapment, enhance intraoperative safety in EMMM, reduce the burden on the surgeon, and facilitate surgery.

## Data Availability

The original contributions presented in the study are included in the article/Supplementary Material, further inquiries can be directed to the corresponding author/s.
